# Activity Engagement and Cognitive Performance Amongst Older Adults

**DOI:** 10.3389/fpsyg.2021.620867

**Published:** 2021-03-11

**Authors:** Alexandria N. Weaver, Susanne M. Jaeggi

**Affiliations:** School of Education, University of California, Irvine, Irvine, CA, United States

**Keywords:** activity engagement, cognitive performance, cognitive reserve, aging, education

## Abstract

Research supporting cognitive reserve theory suggests that engaging in a variety of cognitive, social, and physical activities may serve as protective factors against age-related changes in mental functioning, especially if the activities are cognitively engaging. Individuals who participate in a variety of cognitive activities have been found to be more likely to maintain a higher level of cognitive functioning and be less likely to develop dementia. In this study, we explore the relationship between engaging in a variety of activities and cognitive performance amongst 206 healthy older adults between the ages of 65–85. Age and years of education were found to be the most significant predictors of a global composite representing cognitive performance, consistent with previous work linking these variables to age-related changes in cognition and the cognitive reserve. We interpret these results to suggest that age and education are better predictors of global cognitive performance in older adults than self-reported activity engagement.

## Introduction

Currently, our global population is aging at a fast rate. In 2015, it was estimated that 8.5% of the people worldwide were aged 65 and older, and the number of older individuals was projected to continue to increase (He et al., 2016). By the year 2050, it was estimated that older individuals would represent 16.7% of the worldwide population (He et al., 2016). While an increase in life expectancy is an amazing advancement in humanity, the growing aging population presents various health and economic challenges (Power et al., 2019).

One of those challenges is age-related cognitive decline, which is a common human experience. However, the extent of cognitive decline and cognitive changes can be vastly different between individuals (Salthouse, 2009). One proposed explanation for those individual differences is variability in cognitive reserve. The cognitive reserve can be described as the accumulation of brain resources that are developed through a lifetime of experiences, including the types of activities one engages with, that are used when faced with challenges or damage (Stern, 2002; Cheng, 2016; Cabeza et al., 2018). As an aging society, it is critical to understand whether activity engagement relates to cognitive performance and how it might lead to the development and maintenance of the cognitive reserve.

The literature broadly defines the cognitive reserve as the brain’s ability to compensate in the face of atrophy or challenges, which can occur as the result of diseases such as Alzheimer’s disease, and those that may be experienced as a natural consequence of aging (Cheng, 2016). This ability to compensate, also referred to as the brain reserve (Stern, 2012), is often described as the employment of high functioning neural resources that work harder in order to attempt to maintain similar levels of functioning for brain regions that have suffered damage or are experiencing difficulties (Cheng, 2016). While the exact mechanisms and development/maintenance of the reserve are still debated, the current study operates under the definition of the cognitive reserve as described by Cabeza et al. (2018). They define the reserve as the accumulation of brain resources throughout the lifespan that are well maintained and utilized when necessary (Cabeza et al., 2018). We have adopted this definition of the reserve as previous studies have suggested that brain resources and cognitive performance may be maintained or enhanced as a result of one’s activity engagement (Phillips, 2017; Guiney and Machado, 2018; Lee et al., 2020).

This conceptualization of the reserve attempts to account for individual differences in brain processing and focuses on how experiences such as education, complexity of occupations, and/or engaging in cognitively stimulating leisure activities might serve as protective factors against damage (Barnett et al., 2006; Opdebeeck et al., 2016). The cognitive reserve that may develop and accumulate as a result of years of experiences may be employed and used as a source of compensation, which we define as the neural recruitment that takes place in response to a high cognitive demand that results in some form of enhancement in cognitive performance (Bierre et al., 2017; Cabeza et al., 2018). Thus, this conceptualization assumes that the cognitive reserve is something that can be built upon, changed, and developed with different experiences. As a result of our unique experiences, we have varying amounts of neuronal connections and strengths between those connections across individuals. The cognitive reserve may also help to explain why two individuals who experience similar extents of brain disease or deterioration do not show the same levels of associated cognitive impairment (Barulli and Stern, 2013).

Numerous studies have assessed the association between engaging in a variety of activities such as social, physical and cognitive activities, and cognitive performance amongst older adults (Bielak et al., 2012; Park et al., 2014; Sposito et al., 2015; Poelke et al., 2016; Lee et al., 2019). Engaging in these activities may contribute to the maintenance of the cognitive reserve (Baldivia et al., 2008; Chan et al., 2018) and result in preserved functioning in later life. In addition to activity engagement, other common proxies of the cognitive reserve include education and occupation (Valenzuela and Sachdev, 2006; Stern, 2009).

It is well-known that our social environments, social support, and relationships have considerable benefits to our health. Individuals who have a greater amount of social connections have been shown to have lower mortality risks (Perissinotto et al., 2019). Social support and relationships are also associated with better mental and physical health (Cohen and Herbert, 1996; Seeman et al., 2001; Menec, 2003), as well as cognitive health. For example, studies reported that social engagement, such as volunteer work and visiting with friends and family, was associated with higher levels of cognitive functioning (Krueger et al., 2009; Guiney et al., 2021). However, not all studies report this association (Aartsen et al., 2002). While there are mixed findings on the relationship between social activities and cognitive functioning in older age, social engagement might still contribute to overall health and well-being (Baker et al., 2005).

Engagement in physical activities is also well documented on their benefits to health and well-being. Regular exercise, such as aerobic and anaerobic exercise, has shown to be helpful to manage symptoms of depression (Mata et al., 2012; Schuch et al., 2016). In addition, energy expenditure through physical activity is associated with lower risks of mortality amongst healthy older adults (Manini et al., 2006). Physical activity may also have protective benefits for cognitive functions as higher levels of physical activity in later life are associated with slower age-related cognitive decline (Kawas, 2008; Ku et al., 2012; Stenling et al., 2021). Furthermore, a study by Chang et al. (2010) found that individuals who reported engaging in physical activities during midlife had higher scores for processing speed, memory, and executive function in comparison to individuals who reported no midlife activity. Their results suggest that physical activity during midlife could contribute to the maintenance of cognitive functioning via the cognitive reserve. However, others have not found this relationship (Sposito et al., 2015), and thus, the exact contributions of physical activities to cognitive maintenance are not fully understood. Nonetheless, the potential protective effects of physical activity have been observed through the association between physical activity engagement and lower risks of developing Alzheimer’s disease and related dementias (Buchman et al., 2019; Palta et al., 2019).

Cognitive activities, including leisure-type activities, have also been recognized to play a protective role against cognitive decline. Activities such as reading, writing, and playing board games have been associated with higher cognitive performance (Marquine et al., 2012; Sposito et al., 2015), and a reduced risk of dementia (Verghese et al., 2003). In a study examining the benefits of physical and cognitive activities on simple and complex cognitive tasks amongst young and older adults, the authors found that both physical and cognitive activities were associated with better performance, but cognitive activities were a stronger predictor of complex cognitive tasks, especially amongst older adults (Newson and Kemps, 2006). Their results suggest that both physical and cognitive activities could serve as protective factors against age-related cognitive decline. However, differences in activity type within categories, such as the physical activities riding a bike vs. playing a sport, might influence the cognitive reserve through different pathways (Newson and Kemps, 2006). Although various studies report a relationship between cognitive activities and cognitive functioning, others report conflicting findings. For example, Aartsen et al. (2002) reported that activities across social, physical, and cognitive categories were not related to an enhancement in cognitive performance over a 6-year period. In addition, others examining this relationship have found no association between leisure activities and cognitive functioning in individuals with higher education (Park et al., 2019).

While various activities have been found to be beneficial for cognitive performance in older age, less is known about the potential importance of the specifics of this activity engagement, such as frequency of participation and the variety of activities individuals are engaged in. Specifically, is it enough to maintain cognitive functions by participating in a broad variety of many activities, or is it frequency or repeated engagement in a select few activities that ultimately strengthens and maintains cognitive functions? Frequency of engagement is the most commonly used measure of activity engagement in the literature and has been found to be significantly associated with cognitive performance and is predictive of abilities such as perceptual speed and working memory (WM) (Verghese et al., 2003; Bielak et al., 2012). Nonetheless, Carlson et al. (2012) found an association between participating in a greater assortment of activities and a decreased risk of cognitive impairment, regardless of how cognitively demanding the activities were. In addition, they reported that activity variety (i.e., the participation in many different kinds of activities) was more predictive than frequency of engagement. Similarly, others investigating activity engagement and cognitive performance have found breadth to be predictive of performance over other variables such as time spent on activities (Lee et al., 2020). Yet, others such as Bielak et al. (2019) report conflicting findings, concluding that frequency and breadth seem to have similar associations with cognition.

Given the mixed results in the literature, there is a need to further investigate the potential impact of activity engagement and cognitive performance amongst older adults. The present study aims to answer the following questions using an exploratory, correlational approach: (1) Which activity categories are most predictive of cognitive performance (social, physical, or cognitive)? (2) Does breadth or frequency of activity engagement best predict cognitive performance? Our cognitive outcomes of interests are WM, episodic memory, and processing speed as these are processes that have been shown to be particularly sensitive to the effects of aging (Hartshorne and Germine, 2015; Murman, 2015). Activity engagement and years of education served as proxies for the cognitive reserve. We define frequency of activity engagement as the number of times per week an individual engages with an activity and breadth as the number of activities an individual engages with across a variety of categories (i.e., social, physical, or cognitive).

Our hypothesis for our first research question rests on the assumption that cognitive functioning would be best predicted by engagement in cognitive activities. Cognitively stimulating activities may demand more neural resources associated with this category in comparison with social and physical activities (Fong et al., 2015), which may lead to the maintenance of cognitive abilities. For our second research question, we test whether frequency of activity engagement is more predictive for cognitive functioning than breadth of activity engagement. The reason why frequency of engagement might be more predictive rests on the assumption that once neuroplasticity is initiated by new learning or engagement, frequent engagement and practice with these activities leads to the strengthening of these connections, making them more resilient in the face of challenges (i.e., cognitive decline) (Phillips, 2017). In contrast, as others have demonstrated, variety/variability in activity engagement may be a critical factor that promotes learning and maintenance as well, especially if one engages in novel activities (Bielak et al., 2019; Lee et al., 2020).

## Materials and Methods

### Participants, Data, and Procedure

Data for this analysis are combined from two broader multi-site interventions targeting cognitive and metacognitive skills amongst healthy older adults (Jaeggi et al., 2020). In total, 274 participants were recruited between Southern California and Southeast Michigan. Participants were eligible if they were between the ages of 65–85, had no diagnosis of neurological disorders including mild cognitive impairment, and scored within appropriate ranges of the Mini-Mental State Examination (MMSE) (>24) (Folstein et al., 1975). Additionally, participants were eligible if they were not currently participating in any other cognitive interventions. The present study only utilizes participants’ baseline assessments.

Sixty-eight total participants were excluded from the analysis. Participants were excluded if they were missing data on the activity engagement questionnaire (i.e., they did not respond at all; *n* = 47), the global cognitive performance composite (e.g., were missing all data for a subcomponent of the cognitive performance composite such as all tasks used to assess WM; *n* = 15), or did not meet the screening criteria (*n* = 6). The final analytical sample consisted of 206 participants (mean age = 72.90; SD = 5.43; 74% women). Demographic information of the analytical sample is provided on Table 1. A *post hoc* power analysis was conducted using the software G^∗^Power (Faul et al., 2007). The sample size of 206 was used for the analyses with 11 predictor variables as a baseline. We utilized the recommended effect sizes as follows: small (*f*^2^ = 0.10), medium (*f*^2^ = 0.25), and large (*f*^2^ = 0.40) (Cohen, 1977) with an alpha level of *p* < 0.05. The analyses revealed that the statistical power for this study was 0.87 for detecting a small effect, while the power surpassed 0.99 for detecting a medium to large effect.

**TABLE 1:** Demographic characteristics of participants.

Variable	*n*	*M*	SD	Range
Age	206	72.90	5.43	65–85
Gender				
Female	152			
Male	54			
SES	201	6.77	1.83	3–10
Education (years)	204	16.57	2.53	8–12
Health	202	3.41	0.83	2–5
Anxiety (GAD)	196	1.23	1.85	0–12
Depression (GDS)	194	1.11	1.74	0–9
Well-being (WHOQOL-Old)	193	73.19	11.36	47–100
Cognitive status (MMSE)	206	28.76	1.53	24–30

*Cases were deleted listwise. Socioeconomic status (SES) ranged on a scale from 1 to 10 with higher meaning more well off in comparison to others in the United States. Health was rated on a scale of 1–5, with five meaning above average compared to others their same age. Anxiety (GAD) score of four and below out of 21 indicates no anxiety symptoms. A score of ≥15 indicate severe anxiety. Depression (GDS) score of four and below out of 30 indicates no depressive symptoms. A score ≥10 is indicative of depression. Well-being (WHOQOL) high scores indicate high well-being; scores out of 100 possible points. Cognitive status (MMSE) scores of 24 or greater out of 30 suggest no presence of dementia.*

Prior to completing the assessments, participants were emailed various self-report questionnaires through the online system Qualtrics to capture demographic information, physical and mental health including overall well-being using the World Health Organization Quality of Life group (WHOQOL-Old) (Fang et al., 2012). Participants were additionally screened for general cognitive status as assessed with the MMSE (Tombaugh and McIntyre, 1992), and for depression and generalized anxiety using the Geriatric Depression Scale (GDS) (Yesavage, 1988), and Generalized Anxiety Depression Questionnaire (GAD) (Spitzer et al., 2006). Participants were then asked to come into the lab to complete a battery of assessments that took 2.5 hours on average (maximum of three), to measuring various aspects of cognitive functioning. Because of the extensive testing time, participants took breaks roughly every 45 min or more frequently if requested.

### Assessments

#### Activity Engagement

**FIGURE 1:**
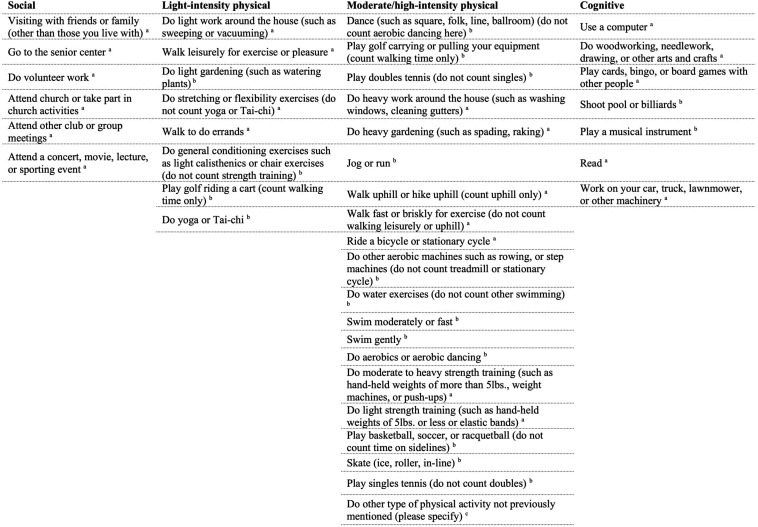
List of items in CHAMPS questionnaire. ^*a*^Indicates activities were included in the analyses, ^*b*^indicates activities were left out of the analyses because 75% or greater of participants reported no engagement in that activity, ^*c*^indicates activity was left out of analyses because it did not contribute additional information.

**FIGURE 2:**
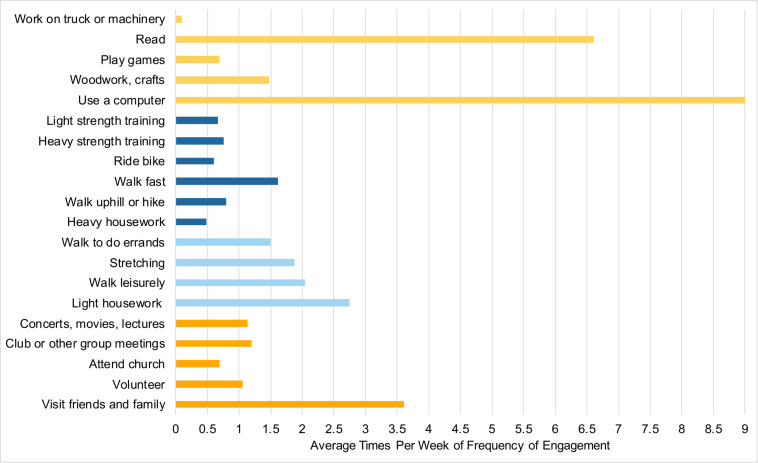
Average times per week of engaging in activities. Cognitive activities are in light orange, moderate/high-intensity physical activities in dark blue, light-intensity physical activities in light blue, social activities are in dark orange.

Participants completed the Community Healthy Activities Model Program for Seniors Physical Activity Questionnaire for Older adults (CHAMPS; Stewart et al., 2001) online through Qualtrics at least 1 week prior to coming into the lab. This 41-item self-report questionnaire assessed their participation, frequency, and duration of various activities within the past 2 weeks. For example, participants were asked if in the previous 2 weeks they visited with friends or family, how often during the week, and for how many hours. For a full list of the items used in the analyses, see Figure 1. The total of activities has shown a test–retest reliability intraclass correlation coefficient (ICC) of 0.56–0.70 (Hekler et al., 2012). Times per week were used as the measure of each individual activity. Activities were excluded from the analysis if 75% or more participants did not engage in the individual activity. The final analysis included 20 activities that were then classified into categories as used in previous studies (Stern and Munn, 2010): cognitive, social, and physical to create category composites. Currently, there is no standardized method to categorize individual activities into social, physical and cognitive categories. Although all of the individual activities presented here could be classified under multiple categories (e.g., dancing could be considered a physical activity and social activity), and the fact that all activities we engage in have some cognitive component, the purpose of this analysis is to explore if there is any relationship between the broad classification of activities and cognitive performance. Specifically, we classified the individual activities into the categories they are most commonly associated with and have a greater emphasis on (e.g., dancing is more commonly considered to be a physical activity over a social one) by relying on previous studies (e.g., Stern and Munn, 2010). In addition, the distinction between light-intensity and moderate/high-intensity physical activities were made as defined by the CHAMPS subscales, and given that previous studies have found differences in cognitive performance based on exercise “intensity” (Hwang et al., 2016). In total, there were five cognitive activities, five social activities, and 10 physical activities. Physical activities were divided into light-intensity (four activities), and moderate/high-intensity (six activities). One question was excluded from the physical activity category (“participate in any other physical activity not mentioned”) because the responses provided did not give any further insight beyond the questions already included. Specifically, participants either reported activities already listed, listed a non-physical activity that was a variant of an activity already included, or did not list an activity at all. See Figure 2 for the average times per week of engagement in activities. The CHAMPS initially captures frequency of each individual activity as an open-ended response. For analysis, the average value was imputed if a range of frequency was reported. To address missing data for frequency of engagement, hot deck imputation was used to keep random variability (Andridge and Little, 2010). Outliers were winsorized to the nearest non-outlying value. Frequency of activity engagement was measured as the sum of frequencies for each individual activity per participant, and breadth was measured as the total number of distinct activities across the three categories (cognitive, social, and physical). Our assessment of activity engagement served as a proxy for the cognitive reserve, along with participants’ self-reported education level.

#### Cognitive Assessments

##### Global cognitive performance

Cognitive performance was measured as a global composite consisting of measures of WM, episodic memory, and processing speed. All cognitive tasks are described in Jaeggi et al. (2020). Each cognitive domain was assessed with three separate tasks in order to capture various aspects of those constructs and to minimize task-specific error variance. Each task was scored individually prior to creating the global cognitive performance composite using z-scores. All cognitive tasks were administered face-to-face in the lab.

*Working memory.* Working memory consisted of three individual tasks. The first task used was the Spatial n-back (Jaeggi et al., 2020) to assess WM updating and was administered via tablet. Stimuli were presented in a moving window that lasted for 1,000 ms with an interstimulus of 2,500 ms. Stimuli were presented one at a time on various locations of a diamond shape composed of circles. The task required indicating whether the presented location of a stimulus was the same as the one presented n trials previously. The stimuli presented could be targets, non-targets, or lures. A lure is an item that resembles the correct response, but is presented at the incorrect n trial. For example, if the participant is required to recall 2-back, the lure is presented 1-back. After one round of 1-back, participants completed three rounds of a 2-back without lures and three rounds with lures. Each round consisted of five target stimuli, 10 + n non-target stimuli, as well as six lures in those rounds that contained lures. The dependent variable was the proportion of hits minus false alarms (pr) across all 2-back trials.

The second WM task was the Sternberg task (Iordan et al., 2018) and was used as a measure of WM maintenance. For this computerized task, participants were presented with a set of uppercase consonant letters (a set size of 4–8) and were given a few seconds to retain them. After their retention period, they were then presented with a lowercase probe letter and had to indicate whether this letter was a part of their initial memory set. Participants completed three blocks of 20 trials. The dependent variable for this task was the average of accurate responses across all trials.

The third WM task was the Symmetry span (Redick et al., 2012) which was used as a variant of a complex WM span, capturing storage and processing. In this computerized task, participants had to indicate whether or not a pattern was symmetrical. After this decision, they were presented with a square that was placed in 1 of 16 locations on a grid. After two to six trials of a symmetry decision and a location on the grid, participants were asked to recall the locations of the squares in order with their computer mouse. The dependent variable was the number of correctly recalled sets.

*Episodic memory.* Episodic memory consisted of three individual tasks. The first episodic memory task used was a verbal Metamemory task (McGillivray and Castel, 2011). Participants were presented with five, 12-word lists and were asked to place a bet between 0 and 10 points after each word on their likelihood of remembering that word in the future. At the end of each list, participants were asked to recall as many words as possible. For every correctly remembered word, their bet for that word was added to their score. For every failure to recall a word, their bet for that word was subtracted from their score. At the end of each list, participants were shown their score before moving onto the next list. Here, the number of correctly recalled words across all lists served as the dependent variable (cf. Parlett-Pelleriti et al., 2019 for a report on the other variables).

The second task used was a measure of visual long-term memory (Perrig et al., 2006). Participants were shown two arrangements of line drawings of objects, patterns, and words on one page similar to Snodgrass and Vanderwart (1980) and were asked to mark all the differences they saw between the two arrangements within 3 min. After about 20 min, participants were asked to perform a surprise recall and report as much as they could from the pictures as well as the differences they found. The total number of correctly recalled items served as the dependent variable.

The third measure used was the Characterization of the Elderly on Daily Activities in the Real-World (CEDAR) (Thomas, 2015). This was an everyday memory task that required participants to take on the role of a fictitious neighbor and complete a series of fictitious errands that involved tasks such as managing medications, finances, and making long-term decisions as a favor for a fictitious character. Accuracy was standardized across subtasks and averaged into a single measure to serve as the dependent variable.

*Processing speed.* Processing speed consisted of three individual tasks. The first used was the D2 (Brickenkamp, 2002). This task consisted of 14 lines of letters presented as either p or d’s, with one to four dashes below and/or above each letter. Participants were given 20 s per line and were asked to cross out any d’s with two dashes as quickly as possible while ignoring the other items. The index of processing speed was the total number of items completed minus any type of error (TN-E).

The second and third tasks consisted of the pattern and letter comparison as used in de Ribaupierre and Lecerf (2006). In the pattern comparison task, participants were asked to decide as quickly as possible if two patterns presented next to each other were identical or not (e.g., QLXVST_QLNSVT) (60 items total). The letter comparison task required the comparison of letter strings (42 items in total). The dependent variables used were total time in seconds it took to complete each of the tasks.

#### Covariates

Covariates used in this analysis included self-reported age, gender, socio-economic status (SES) (Adler et al., 2000), years of education, and physical health. To report SES, participants were shown a ladder with 10 rungs to represent where people stand in the United States. The top of the ladder (labeled number 10) represented people with the most money, education, and respected jobs. The bottom of the ladder (labeled number 1) represented people with the least money, education, and respected jobs. Participants were asked to place themselves on the ladder (between 1 and 10) of where they currently stood relative to others in the United States. To report physical health, participants were asked to compare their physical health to others their own age on a scale of 1 much worse than average, to 5 much better than average.

#### Analytical Approach

Data was analyzed using IBM SPSS Statistics Version 25. For the analyses, a series of multiple regressions were conducted. To address the first hypothesis of which activities were predictive of global cognitive performance, four separate hierarchical regressions (one for each activity type) were conducted with global cognitive performance as the outcome variable. For each hierarchical regression, demographic variables; age, gender, SES, year of education, and self-reported health were entered at step one, and the activity categories (social, light-intensity physical, moderate/high-intensity physical, cognitive) were entered at step two.

To address the second hypothesis of breadth or frequency of activity engagement predicting global cognitive performance, two hierarchical regressions were conducted. Just as in the previous regressions, demographic variables were entered at step one and then breadth or frequency of activity engagement was entered at step two.

Exploratory regression analyses were used to investigate whether certain categories were more predictive of the subcomponents of the global cognitive performance composite (i.e., WM, episodic memory, or processing speed). The data underwent assumptions testing appropriate for multiple regressions and met the criteria of linearity, multicollinearity, and homoscedasticity. However, the activity categories (social, light-intensity physical, moderate/high-intensity physical, cognitive) as well as frequency of activity engagement, violated the assumption of normality. Nonetheless, we proceeded with this choice of method as regressions have been found to be robust to this violation (Schmidt and Finan, 2018).

## Results

### Activity Categories as Predictors of Global Cognitive Performance

For correlations, see Supplementary Table 1 in Supplementary Material. See Table 2 for hierarchical regression results. Overall, none of the activity categories were predictive of global cognitive performance. However, age and education were significant predictors of global cognitive performance.

**TABLE 2:** Hierarchical regression results for activity categories as predictors of global cognitive performance.

	Social		Light-intensity		Moderate/high-intensity		Cognitive	
				
Variable	Step 1	Step 2	Step 1	Step 2	Step 1	Step 2	Step 1	Step 2
Age	−0.34*** (0.06)	−0.35*** (0.06)	−0.37*** (0.06)	−0.37*** (0.06)	−0.33*** (0.06)	−0.33*** (0.06)	−0.33*** (0.06)	−0.33*** (0.06)
Gender	0.00 (0.72)	−0.03 (0.75)	0.01 (0.70)	0.00 (0.70)	0.01 (0.71)	0.01 (0.72)	0.00 (0.72)	0.00 (0.72)
SES	−0.10 (0.18)	−0.13 (0.19)	−0.11 (0.18)	−0.13 (0.18)	−0.11 (0.18)	−0.11 (0.18)	−0.10 (0.19)	−0.10 (0.19)
Education	0.20* (0.13)	0.20* (0.13)	0.18* (0.12)	0.18* (0.12)	0.20* (0.13)	0.20* (0.13)	0.20* (0.13)	0.20* (0.13)
Health	0.02 (0.38)	0.11 (0.38)	0.08 (0.38)	0.08 (0.38)	0.05 (0.38)	0.05 (0.47)	0.03 (0.38)	0.03 (0.38)
Social activities		0.11 (0.38)						
Light-intensity physical activities				0.11 (0.34)				
Moderate/high-intensity physical activities						−0.01 (0.47)		
Cognitive activities								−0.01 (0.19)
Constant	15.35** (5.02)	15.98** (5.03)	16.82*** (4.83)	16.34*** (4.83)	14.54** (5.00)	14.54** (5.01)	14.53** (5.05)	14.72** (5.20)
*N*	148	148	148	148	145	145	147	147
*R*^2^	0.15	0.16	0.17	0.18	0.15	0.15	0.14	0.16
Δ*R*^2^	0.15***	0.01	0.17***	0.01	0.15***	0.00	0.11**	0.00

For the variable gender, 0 = male, 1 = female. Standardized coefficients are reported and standard errors are in parentheses. Cases were deleted listwise. *p < 0.05, **p < 0.01, ***p < 0.001.

### Activity Frequency and Breadth as Predictors of Global Cognitive Performance

See Table 3 for hierarchical regression results. Overall, activity frequency and breadth were not found to be predictive of global cognitive performance, but age and education remained to be significant predictors of global cognitive performance.

**TABLE 3:** Hierarchical regression results for breadth and frequency of activity engagement as predictors of global cognitive performance.

	Breadth		Frequency	
		
Variable	Step 1	Step 2	Step 1	Step 2
Age	−0.37***(0.06)	−0.37***(0.06)	−0.34***(0.06)	−0.34***(0.06)
Gender	0.02(0.70)	0.03(0.71)	0.01(0.71)	0.01(0.72)
SES	−0.11(0.18)	−0.11(0.18)	−0.10(0.18)	−0.10(0.19)
Education	0.20*(0.13)	0.20*(0.13)	0.19*(0.13)	0.18*(0.13)
Health	0.05(0.38)	0.05(0.38)	0.08(0.39)	0.08(0.39)
Breadth		−0.02(0.13)		
Frequency				0.05(0.02)
Constant	16.57**(4.89)	16.86**(5.04)	14.43***(5.02)	14.12***(5.06)
*N*	145	145	141	141
*R*2	0.18	0.18	0.15	0.15
Δ*R*2	0.18***	0.00	0.15***	0.00

For the variable gender, 0 = male, 1 = female. Standardized coefficients are reported and standard errors are in parentheses. Cases were deleted listwise. *p < 0.05, **p < 0.01, ***p < 0.001.

### Exploratory Analyses of Cognitive Composite Subcomponents

Hierarchical regressions were conducted to examine the relationship between the activity categories and each subcomponent of the global cognitive composite (i.e., WM, episodic memory, and processing speed). None of the activity categories were found to predict any of the cognitive subcomponents.

## Discussion

Previous research suggests that engaging in a variety of activities may provide protective benefits against the effects of age-related cognitive decline as these types of activities may contribute to one’s cognitive reserve by building new and strengthening existing neuronal connections (Newson and Kemps, 2006; Sposito et al., 2015). In the present study, we examined whether social, physical, and cognitive activities were predictive of global cognitive performance, and furthermore, if breadth or frequency of activity engagement was predictive of cognitive performance utilizing a series of hierarchical regressions. Based on previous studies (Verghese et al., 2003; Bielak et al., 2012; Marquine et al., 2012; Sposito et al., 2015), we hypothesized that cognitive activities and frequency of activity engagement would be predictive of global cognitive performance.

In contrast to previous findings (Singh-Manoux et al., 2003; Verghese et al., 2003; Bielak et al., 2012; Carlson et al., 2012; Lee et al., 2019), our results indicate that none of the activity categories or breadth/frequency of activity engagement were predictive of global cognitive performance. However, age and years of education significantly predicted cognitive performance. Exploratory analyses examined if activity categories were predictive of any of the subcomponents of the global cognitive performance composite (i.e., WM, episodic memory, and processing speed), however, none were predictive of the cognitive subcomponents.

Our finding that age and education were predictive of cognitive performance is in line with previous research on cognitive aging, and they illustrate the importance of education as one of the key contributing factors to the cognitive reserve (Valenzuela and Sachdev, 2006; Baldivia et al., 2008; Thow et al., 2017). Global cognitive performance got worse as a function of higher age reflecting age-related cognitive decline, whereas higher education was associated with better performance. Importantly, age and education were predictive of global cognitive performance across all hierarchical regression models.

Although activity engagement was our primary variable of interest, education is often used as the primary indicator for the cognitive reserve (Valenzuela and Sachdev, 2006; Stern, 2009). Previous studies have consistently observed a relationship between education and cognitive health (Tucker-Drob et al., 2009; Sattler et al., 2012; Farfel et al., 2013), which has been interpreted in that education might facilitate the development of cognitive strategies as well as help maintain cognitive performance, especially if education is pursued into late adulthood (Thow et al., 2017). Other studies that have found this relationship have suggested that higher levels of education might lead to various lifestyle choices that could impact health (Hooren et al., 2007). An additional explanation may be that more education may lead to mental stimulation throughout life that results in the maintenance of cognitive functions and is likely that individuals with more education might have occupations that involve more mental stimulation (Hooren et al., 2007; Baldivia et al., 2008). Unfortunately, we do not have data collected in our population that could speak to this hypothesis. However, our sample has a relatively high level of education on average albeit with some variability (range of 8–20 years, *M* = 16.57), which may speak to our finding of higher education predicting better performance.

While various studies report a positive association between activity engagement and cognitive performance (Park et al., 2014; Lee et al., 2019, 2020), findings have been inconsistent across studies, especially with regards to the type of activities assessed, and the constructs of cognitive functioning they are associated with (Parisi et al., 2009; Poelke et al., 2016; Bielak, 2017). Our results do not seem to provide more clarity to the current literature on activity engagement and cognitive performance. It is possible that the variation in results can be attributed to differences in how cognitive performance is defined and assessed, differences in measurement and classification of activity engagement and activity type, the age range of the population, as well as participants’ overall level of engagement. One potential reason for our findings might be the fact that we relied exclusively on the CHAMPS questionnaire to assess activity engagement. The CHAMPS questionnaire was originally created as a measure of physical activity and caloric expenditure. As a result, there was an overrepresentation of physical activities for participants to select from than what we categorized as social and cognitive. As such, our measure of activity engagement might not fully capture the various activity categories as well as activities one could engage with within those categories, including breadth and frequency. In addition, the questionnaire asks participants to report if they have engaged with these activities in the previous 2 weeks, and it is possible that participants may have been reporting engagement in activities that they do not regularly engage with. For various activities in the questionnaire, we cannot conclude that participants engage with these activities regularly and consistently, and furthermore, we have no knowledge about how many years they might have participated in these activities. It is possible that more long-term and consistent engagement in activities might be related to cognitive performance in later life and a more long-term measure of activity engagement might better capture this (Chang et al., 2010; Chan et al., 2018). However, the interpretation of the literature is challenging because studies have differed in their specifications of the time interval of activity engagement, ranging from no specification (Ihle et al., 2017), to indicating once per month to daily engagement (Krell-Roesch et al., 2019). Despite those variations in timing, previous studies have generally reported a positive relationship with cognitive performance. Although it seems to be a valid assumption that more long-term engagement may reflect cognitive reserve more adequately, activity engagement measurement with shorter time interval specifications have also reported positive relationships with cognition, even though our results do not. Thus, it is possible that activity engagement as assessed here does not have a strong effect on the specific areas of cognition we measured.

Several limitations of this study should be noted. The first limitation pertains to the population recruited, which was generally high-functioning and likely not representative of the greater population. People were recruited via flyering and through databases participants register for to be contacted about participating in research studies. As such, participants self-selected to participate in this memory study. It is possible that individuals who are concerned with their cognitive functioning with aging may already proactively engage in a variety of lifestyle activities aimed at maintaining or increasing their cognitive performance, including generally participating in memory-related studies. Indeed, participants in this study presented to be a highly engaged group as there was little variation in individuals who engaged in a lot of activities vs. individuals who engaged in fewer activities. Participants reported that they were generally in very good health in comparison to others their age. This could result in greater or more long-term engagement with activities that could contribute to the maintenance of cognitive functions, although we only measured activities they engaged with in the previous 2 weeks upon joining the study. Because there was little variation between individuals, we might not have been able to detect a difference in cognitive performance based on activity engagement.

As previously mentioned, the measurement of activity engagement used here may not be an ideal and comprehensive measure of activity engagement. The CHAMPS questionnaire asks participants to report whether or not they engage in an activity and the hours and times per week spent on those activities. It is possible that the data may not be representative or accurate. Previous studies have suggested that individuals may under- or over-report their time spent engaging in activities (Salthouse et al., 2002; Parisi et al., 2009), which could have been even further exacerbated by the fact that we implemented a retrospective assessment that relied on participants’ memory functions. If participants under or over reported their activity engagement, then the missing data imputation method may have only further distorted the data.

## Conclusion

In conclusion, the overall goal of this study was to examine the relationship between activity engagement and cognitive performance amongst older adults. We found that only age and education were predictive of cognitive performance, not activity category, activity breadth, or frequency of engagement. Our results are consistent with previous work demonstrating that education plays a significant role in contributing to the cognitive reserve, which is associated with higher cognitive performance. Our results further suggest that education may be a better predictor of cognitive functioning in older age than one’s activity engagement, potentially reflecting lifestyle choices that have long-term impacts on cognitive health. However, our findings should be interpreted with caution. Although we did not find a relationship between activity engagement and overall cognitive performance, it does not mean that one’s activity engagement does not contribute to cognitive functioning. Engaging in activities one enjoys can have positive effects on overall well-being that may impact health, which might ultimately affect cognitive functioning as well (Aartsen et al., 2002; Baker et al., 2005). Our study is in line with this hypothesis, as we found positive correlations between well-being and social activities (*r* = 0.37, *p* < 0.01), as well as with frequency (*r* = 0.28, *p* < 0.05) and breadth of engagement (*r* = 0.22; *p* < 0.05) (see Supplementary Table 1 in Supplementary Material). Future studies should consider using more holistic measurements of activity engagement, inquire about activity engagement over one’s lifetime, and consider including a broader range of cognitive measures. Additional longitudinal and interventional research is also necessary to determine a causal relationship between one’s activity engagement and cognitive performance in older age.

## Data Availability Statement

The raw data supporting the conclusions of this article will be made available by the authors, without undue reservation.

## Ethics Statement

The studies involving human participants were reviewed and approved by IRB at UCI. The patients/participants provided their written informed consent to participate in this study.

## Author Contributions

AW took the lead in concept development, data analysis, and wrote the first draft of the manuscript. SMJ assisted with data analysis and edited of the manuscript. Both authors contributed to the article and approved the submitted version.

## Conflict of Interest

SMJ has an indirect conflict of interest with the MIND Research Institute, whose interests are related to this work. The remaining author declares that the research was conducted in the absence of any commercial or financial relationships that could be construed as a potential conflict of interest.
