# Supplementary material for: Activity Engagement and Cognitive Performance Amongst Older Adults
Source: Front Psychol. 2021 Mar 11;12:620867. doi: 10.3389/fpsyg.2021.620867 (PMC7990770; doi:10.3389/fpsyg.2021.620867)
Supplement: Supplementary file 1 [file Table_1.DOCX]

| Table S1  *Correlations for Study Variables* | | | | | | | | | | | | | | | |
| --- | --- | --- | --- | --- | --- | --- | --- | --- | --- | --- | --- | --- | --- | --- | --- |
| Variable | 1 | 2 | 3 | 4 | 5 | 6 | 7 | 8 | 9 | 10 | 11 | 12 | 13 | 14 | 15 |
| 1. Age | - |  |  |  |  |  |  |  |  |  |  |  |  |  |  |
| 2. Gender | -.24** | - |  |  |  |  |  |  |  |  |  |  |  |  |  |
| 3. SES | 0.03 | -0.11 | - |  |  |  |  |  |  |  |  |  |  |  |  |
| 4. Education (years) | 0.13 | -0.1 | 0.14 | - |  |  |  |  |  |  |  |  |  |  |  |
| 5. Health | 0.06 | -0.06 | -0.08 | 0.1 | - |  |  |  |  |  |  |  |  |  |  |
| 6. Anxiety (GAD) | 0.03 | 0.01 | 0.11 | -0.09 | -0.17 | - |  |  |  |  |  |  |  |  |  |
| 7. Depression (GDS) | -0.01 | -0.16 | 0.08 | -0.11 | -0.15 | .51** | - |  |  |  |  |  |  |  |  |
| 8. Well-being (WHOQOL-Old) | 0.05 | .21* | .24** | -0.12 | -0.1 | -.18* | -.28** | - |  |  |  |  |  |  |  |
| 9. Cognitive impairment (MMSE) | -0.08 | 0.04 | -0.09 | .33** | 0.13 | -0.12 | -0.08 | .19* | - |  |  |  |  |  |  |
| 10. Global cognitive performance | -.32** | 0.08 | -0.1 | 0.1 | 0.09 | -0.05 | -.22* | 0.03 | 0.13 | - |  |  |  |  |  |
| 11. Social activities | 0.07 | .23* | 0.13 | 0.04 | 0.03 | -0.06 | -.28** | .37** | 0.01 | 0.08 | - |  |  |  |  |
| 12. Light-intensity physical activities | -0.03 | 0.07 | 0.11 | -0.02 | -0.05 | 0.11 | 0.12 | 0.12 | -0.09 | 0.11 | 0.17 | - |  |  |  |
| 13. Moderate/high-intensity physical activities | 0.01 | -0.09 | 0.08 | 0.05 | 0.09 | 0.09 | 0.0 | 0.12 | 0.04 | -0.01 | 0.16 | .34** | - |  |  |
| 14. Cognitive activities | -0.14 | 0.0 | 0.12 | 0.12 | -0.09 | 0.09 | .21* | 0.13 | 0.17 | 0.02 | 0.14 | 0.14 | .23* | - |  |
| 15. Frequency | -0.07 | 0.07 | .18* | 0.1 | -0.04 | 0.1 | 0.08 | .28** | 0.09 | 0.07 | .51** | .57** | .59** | .78** | - |
| 16. Breadth | -0.04 | 0.16 | 0.12 | -0.13 | 0.0 | 0.15 | -0.15 | .22* | -0.07 | -0.05 | .35** | .29** | .54** | 0.15 | .46** |
| *Note.* *N* = 128. Cases were deleted listwise. Socioeconomic status (SES); anxiety (GAD); depression (GDS); well-being (WHOQOL-Old); cognitive impairment (MMSE); global cognitive performance is a composite of WM, episodic memory, and processing speed. Social, light-intensity physical, moderate/high-intensity physical, and cognitive activities are the composite activity categories. Frequency is a composite of the sum of frequencies for each individual activity per participant. Breadth is a composite of the total number of distinct activities across the three categories.  **p* <. 05, ***p* < .01, ****p* < .001 | | | | | | | | | | | | | | | |
